# The ankle ligament reconstruction-return to sport after injury (ALR-RSI) is a valid and reproducible scale to quantify psychological readiness before returning to sport after ankle ligament reconstruction

**DOI:** 10.1007/s00167-020-06020-6

**Published:** 2020-04-30

**Authors:** François Sigonney, Ronny Lopes, Pierre-Alban Bouché, Elliott Kierszbaum, Aymane Moslemi, Philippe Anract, Alexandra Stein, Alexandre Hardy

**Affiliations:** 1 Orthopaedic Department, Cochin University Hospital, AP-HP Paris, Paris Descartes University, 27 rue du Faubourg Saint-Jacques, 75014 Paris, France; 2 Orthopaedic Department, Pied Cheville Nantes Atlantique (Santé Atlantique Et Clinique Bretéché), Nantes, France; 3 Clinique du Sport, 36 boulevard Saint-Marcel, 75005 Paris, France

**Keywords:** ALR-RSI, Ankle, Ligament reconstruction, Psychological

## Abstract

**Purpose:**

Chronic ankle instability is the main complication of ankle sprains and requires surgery if non-operative treatment fails. The goal of this study was to validate a tool to quantify psychological readiness to return to sport after ankle ligament reconstruction.

**Methods:**

The form was designed like the anterior cruciate ligament-return to sport after injury scale and “Knee” was replaced by the term “ankle”. The ankle ligament reconstruction-return to sport after injury (ALR-RSI) scale was filled by patients who underwent ankle ligament reconstruction and were active in sports. The scale was then validated according to the international COSMIN methodology. The AOFAS and Karlsson scores were used as reference questionnaires.

**Results:**

Fifty-seven patients (59 ankles) were included, 27 women. The ALR-RSI scale was strongly correlated with the Karlsson score (*r* = 0.79 [0.66–0.87]) and the AOFAS score (*r* = 0.8 [0.66–0.87]). A highly significant difference was found in the ALR-RSI between the subgroup of 50 patients who returned to playing sport and the seven who did not: 68.8 (56.5–86.5) vs 45.0 (31.3–55.8), respectively, *p* = 0.02. The internal consistency of the scale was high (*α* = 0.96). Reproducibility of the test–retest was excellent (*ρ* = 0.92; 95% CI [0.86–0.96]).

**Conclusion:**

The ALR-RSI is a valid, reproducible scale that identifies patients who are ready to return to the same sport after ankle ligament reconstruction. This scale may help to identify athletes who will find sport resumption difficult.

**Level of evidence:**

III.

## Introduction

Ankle sprains are extremely frequent; they represent 15–20% of all sport-related injuries. The frequency of diagnosed ankle sprains is 6000 cases per day in France and 24,000 cases per day in the United States: they represent 4–7% of emergency department consultation [14]. Functional treatment (Rest, Ice, Compression, and Elevation) is the standard reference for acute ankle sprains. In most cases, it is sufficient for a complete recovery. However, 20% of patients develop chronic lateral ankle instability (CLAI) [8, 16], some require surgical treatment, particularly those who are young and athletic.

Anatomic repairs by retentioning and directly suturing the anterior talofibular ligament (ATFL) are accepted to be the gold standard for treatment of CLAI [6, 10] although arthroscopic anatomical ligament reconstruction is becoming more and more popular. Techniques are developed to perform anatomical reconstruction of the anterior talofibular ligament (ATFL) and the calcaneofibular ligament (CFL) with a tendon graft (gracilis) using an all-arthroscopic approach [10–12, 19]. Ankle arthroscopy is increasingly used to treat CLAI, and offers an opportunity to assess and treat any associated injuries [10, 18, 30] with reduced iatrogenesis [20, 21].

After sustaining ankle injury, the most important issues for athletes are to recover as fast as possible and to return to sport at their initial performance level. It has been noted that many patients after surgery do not return to sport activities at the same level as prior to the injury, even though they have recuperated good functional results [22, 25, 29].

Indeed, studies have shown that athletes go through different stages after an injury, so they must be not only physically, but also psychologically ready to return to the same sport at the same level [7, 26].

Scales to analyze the psychological readiness to return to sport after reconstructive surgery have been developed for other athletic injuries. Webster et al. [31] developed the anterior cruciate ligament-return to sport after injury (ACL-RSI) a scale of 12 items, to quantify the psychological readiness of athletes to return to sport following surgical ACL reconstruction.

The scale measures athlete’s emotions, confidence in performance, and appraisal in relation to return to sport. Gerometta et al. [9] adapted the score, to the return to sport after shoulder instability (treated by surgery or conservative management), with the shoulder instability-return to sport after injury (SIRSI) scale.

The main purpose of this study was to propose and to validate a similar tool to quantify psychological readiness to return to sport after ankle ligament reconstruction.

## Material and method

This study was approved by an Institutional Review Board (CPP IDF III, Hôpital Tarnier-Cochin) (Ref. CPP: 3730-NI; Ref CNRIPH: 19.06.17.35910). Informed consent was obtained from each patient.

### Study participants

This study included patients who were active in sports and underwent ankle ligament reconstruction between January 2016 and May 2017. Patients were excluded if they did not practice sport.

An arthroscopic anatomical reconstruction of the lateral ankle ligaments [11, 12, 19, 23] was performed in all patients.

Fifty-seven patients were included (59 ankles), 27 (47.4%) women and 30 men (Table 1) with a median of 3.0 (2.5; 3.7) years after ankle ligament reconstruction. All patients were athletes who practiced: in competition: 29 ankles (49.2%), as a regular leisure activity: 26 ankles (44.0%) or an occasional leisure activity: four ankles (6.8%). Arthroscopic anatomic reconstruction with tendon grafting was performed in all patients (Table 1).

**Table 1:** Participants

Parameters	Values	*N*	Statistics
Sex	Women	27	47.4%
	Men	30	52.6%
Follow-up (years)		59	3.0 (2.5; 3.7)
ALR-RSI total		59	64.4 (10.0; 100.0)
Karlsson total		59	85.2 (25.0 ;100.0)
AOFAS total		59	81.7 (29.0; 00.0)
Sport recovery	No	7	12.1%
	Yes	52	88.1%
If yes	Sport change	14	27.0%
	Same sport, inferior level	12	23.0%
	Same sport, same level	26	50.0%
Sport level	Competition	29	49.2%
	Casual leisure level	4	6.8%
	Regular leisure level	26	44.0%
Sport	Athletics	1	1.7%
	Badminton	1	1.7%
	Basketball	7	11.9%
	Running	7	11.9%
	Dance	3	5.1%
	Horse riding	1	1.7%
	Fitness	1	1.7%
	Soccer	11	18.6%
	Gymnastic	2	3.4%
	Handball	5	8.5%
	Multiple	11	18.6%
	Rugby	2	3.4%
	Archery	1	1.7%
	Triathlon	1	1.7%
	Volleyball	1	1.7%
	Walk	3	5.1%
	Table tennis	1	1.7%

### Ankle ligament reconstruction-return to sport after injury (ALR-RSI) scale

The ALR-RSI scale is adapted from the ACL-RSI scale. The French version of the ACL-RSI scale has been shown to be valid [5], following international guidelines for the cross-cultural adaptation of self-administered questionnaires [4].

**Fig. 1:**
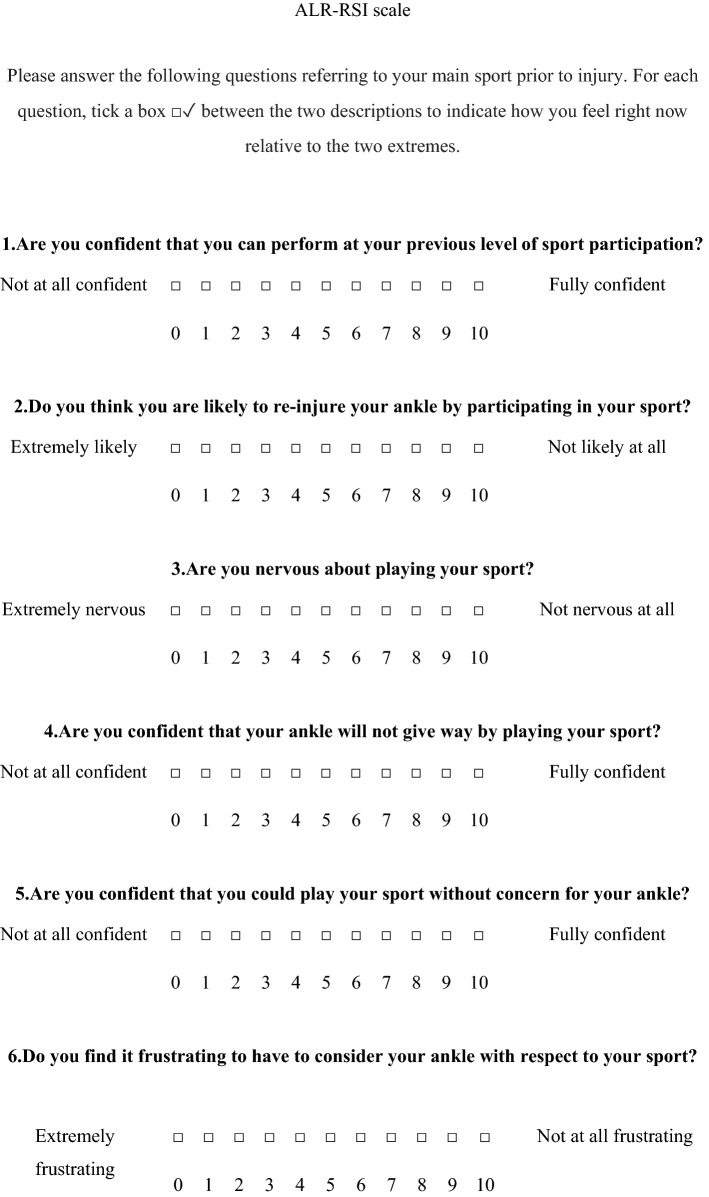

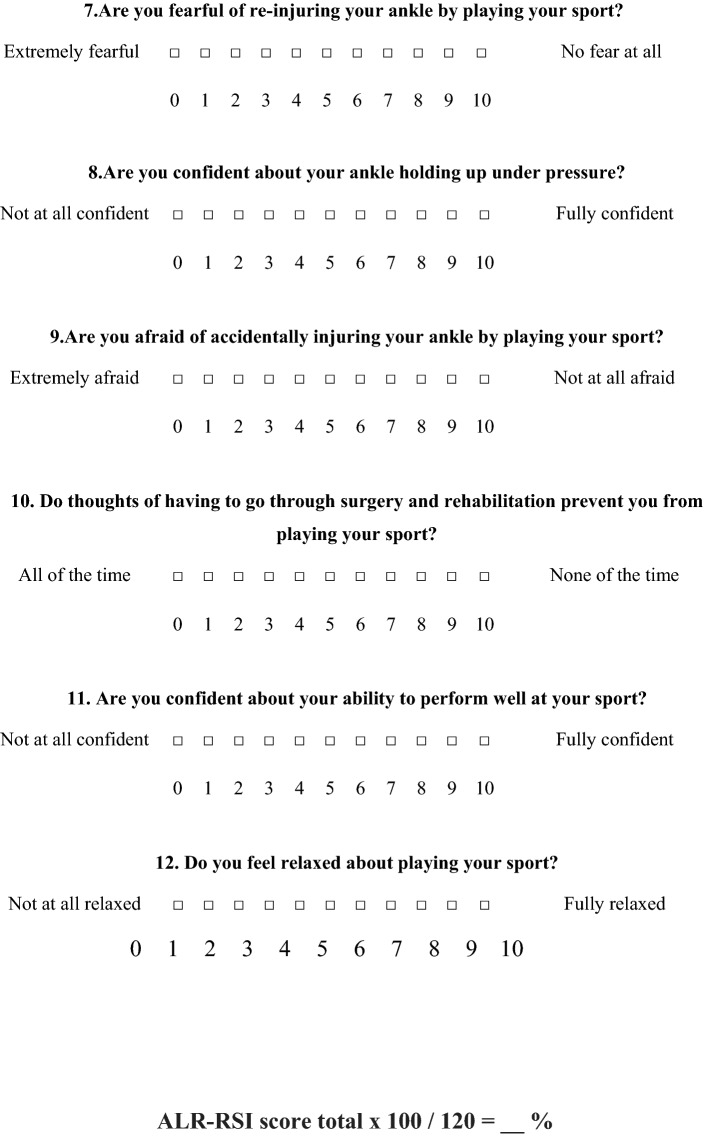
ALR-RSI scale

The term “knee” has been replaced by the word “ankle” in the questionnaire (question 2, 4, 5, 6, 7, 8, and 9). For example, the question 2: “Do you think you are likely to re-injure your knee by participating in your sport?” was replaced by “Do you think you are likely to re-injure your ankle by participating in your sport?” (Fig. 1).

### Validity and reproducibility of ALR-RSI

The final version was validated according to COSMIN international guidelines (COnsensus-based Standards for the selection of health status Measurement Instruments) [24]

The reference scales used were the American Orthopedic Foot & Ankle Society (AOFAS) score [15] and the Karlsson score [27].

Patients were contacted, after giving their consent, they answered a self-administered questionnaire, which included the ALR-RSI scale, the AOFAS score, the Karlsson score and questions regarding their return to sport. The ALR-RSI was completed twice at a 15-day interval.

### The ALR-RSI

The ALR-RSI scale, like the original version of the ACL-RSI, was based on three components that have shown significant association with the return to sport: emotions, confidence in one’s performance and evaluation of risk [26]. The ALR-RSI includes 12 questions with an 11-point Likert scale in the form of blocks to be ticked from 0 to 10. The total score was calculated by adding up the values of the 12 answers then dividing the result by 1.2 to obtain a percentage. High scores corresponded to a positive psychological response.

### Statistical analysis

The R software (version 3.5.0 spotted at the URL https://www.R-project.org) was used to perform the statistical analyses. A sample size of 55 produces a two-sided 95% confidence interval with a width smaller than 0.28 when the estimate of Spearman's rank correlation is above 0.750. Continuous quantitative variables were described by their mean, minimum and maximum. To describe the dichotomous variables, the number of events and their percentage were used. The Karlsson score and the AOFAS score were between 0 (very poor) and 100 (excellent). Construct validity was tested between the ALR-RSI, the total Karlsson score and its different subitems, and the AOFAS score by the Spearman coefficient. The Spearman correlation coefficient r was considered to be “strong” (*r* > 0.5), «moderate» (0.5 < *r* < 0.3) or “strong” (0.3 < *r* < 0.1). Discriminant validity was tested between the group of patients “that had returned to do sport” and the group “that had not returned to do sport” by the Mann and Whitney test. The internal consistency was evaluated by the correlation between the 12 items of the ALR-RSI, by the Cronbach alpha coefficient. Correlation among the items of the questionnaire was considered to be “excellent” if *α* ≥ 0.90. Reliability was evaluated on the *ρ* intraclass correlation coefficient (ICCC). Reproducibility was considered to be “excellent” (*ρ* > 0.75), “good” (0.75 < *ρ* < 0.40) or “weak” (*ρ* < 0.40). Feasibility was estimated by the percentage of missing responses and the ceiling and floor effects, corresponding to a percentage of patients who attained the minimum score (0) or the maximum score (10) for each question. *p* < 0.05 was considered to be significant. The threshold of significance retained was 5% for a power of 80% and an alpha risk of 5%.

## Results

### Return to sport

Fifty (87.7%) patients returned to sport: 24 patients (42.1%) returned to the same sport as before the injury at the same level, 13 patients (22.8%) at an inferior level, and 13 (22.8%) changed sport.

### Construct validity

The ALR-RSI was strongly (*r* > 0.50) and significantly correlated to the Karlsson score: *r* = 0.79 [0.66–0.87] and the AOFAS score: *r* = 0.8 [0.66–0.87] (Tables 2 and 3).

**Table 2:** Correlation the ALR-RSI score and the Karlsson score

Coefficient	ALR-RSI (/100)	Karlsson tot (/100)	Pain (/36)	Other symptoms (/28)	ADL (/68)	Sport (/20)	ARQL (/16)
	66.7 (47.5–85.8)	91.1 (80.1–96.7)	32.0 (28.0–35.0)	25.0 (20.0–27.0)	67.0 (63.5–68.0)	17.0 (13.5–20.0)	12.0 (9.0–14.5)
Spearman		0.79 [0.66–0.87]	0.70 [0.50–0.81]	0.60 [0.42–0.74]	0.65 [0.45–0.78]	0.82 [0.69–0.90]	0.79 [0.66–0.87]

**Table 3:** Correlation the ALR-RSI score and the AOFAS score

Coefficient	ALR-RSI (/100)	AOFAS tot (/100)
	66.7 (47.5–85.8)	88.0 (74.0–94.0)
Spearman		0.8 [0.66–0.87]

### Discriminant validity

A highly significant difference was found in the ALR-RSI between the subgroup of 50 patients who returned to playing sport and the seven who did not: 68.8 (56.5–86.5) vs 45.0 (31.3–55.8), respectively, *p* = 0.02

### Feasibility

None of the answers were missing.

The floor effect, corresponding to the patients with the lowest score for each question, varied, between 0 and 1.7%, and the ceiling effect, corresponding to the percentage with the highest score for each question varied between 6.7 and 33.8%.

### Reliability

**Fig. 2:**
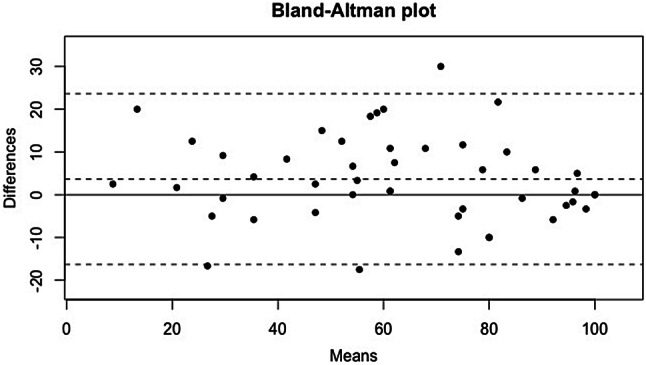
Reproducibility of the ALR-RSI score with the test–retest: Bland–Altman plot

Reliability was evaluated on the *ρ* intraclass correlation coefficient (ICCC). The reproducibility was “excellent” with a *ρ* intraclass correlation coefficient of 0.92 [0.86–0.96]. The median ALR-RSI score was 66.3 (45.6–85.8) when it was filled for the first time and 57.1 (38.1–79.0) the second time (Fig. 2, Table 4).

**Table 4:** Reproducibility of the ALR-RSI score with the test–retest

Coefficient	ALR-RSI 1 (/100)	ALR-RSI 2 (/100)
	66.3 (45.6–85.8)	57.1 (38.1–79.0)
ICC		*ρ *= 0.92 [0.86–0.96]

### Internal consistency

The internal consistency of the scale measuring the strength of the correlation between the 12 items was “excellent” with a Cronbach alpha coefficient of 0.96.

## Discussion

The principal findings of the study were that the ALR-RSI represented a valid and reliable instrument, that can identify patients who are psychologically ready to return to the same sport, after ankle ligament reconstruction.

Patients selected for the surgery are usually young and athletic, and the most important goal of ankle ligament reconstruction is to enable them to return to their prior level in sports [14, 25, 29]. After well-conducted surgery and physical therapy, some patients experience failure or a decrease in their performance, without physically objectifiable reasons. Indeed, patients have to be ready not only physically, but also psychologically, to resume sports after a surgical intervention.

At a median 3.0 (2.5; 3.7) years’ follow-up, return to sport was possible for 87.7% of patients in this study. Those results are similar to what is reported in the literature. In a prospective study [18], the rate of return to the same level of sports following arthroscopic treatment of CLAI was 90% in the repair group and 80% in the reconstruction group, for recreational athletes and 73% after repair compared to 48% after reconstruction, for competitive athletes. Maffulli et al. [22] published the long-term results (9 years) on athletes, following a Bröstrom procedure, 58% were able to return to their preinjury level, 16% at a lower level, and 26% abandoned active sport participation. Nery et al. [25] published also the long-term results (9.8 years) following arthroscopic Bröstrom Gould procedure, 26 (86.7%) of the 30 active patients practiced sport at the same preoperative level, 3 (10%) had changed to a lower level (the AOFAS score at the last follow-up for these three patients was 86, 97 and 44), and 1 (3.3%) had given up sport (AOFAS score at the last follow-up: 87). Therefore, three of four patients, whose sports level decreased, had an excellent AOFAS score.

The different functional scores concerning the ankle, the Karlsson score and the AOFAS score were chosen because they are the most commonly used.

However, their clinical value is not sufficient, for giving patients the permission to return to sport. They do not always correspond to the actual recovery of their athletic performance, because they inform on an objective state of ankle function without taking into consideration the psychological state. Their actual sport capacities were analyzed to circumvent that issue. The patients were specifically asked if they have returned at the same level as prior to the injury, at a lower level or abandoned a specific sport activity. This allows better insight into their real recovery.

A systematic review published by Ardern et al. [2], looked into the psychological factors associated with returning to sport following injury. They showed that motivation, confidence and low fear were associated with an increased likelihood of returning to the preinjury level. On the other hand, fear stood out as the strongest negative emotion preventing a rapid and full return to sport. It has been shown in the context of ACL surgery [3] that patients with positive psychological responses before surgery and at the start of recovery were associated with a better return to sport, suggesting that attention to recovery psychological in addition to physical recovery could be justified.

Clinical screening for inappropriate psychological responses in athletes can help clinicians identify athletes at risk of not returning to their sport level.

The use of a questionnaire with numeric answers makes it possible to simplify the responses, and to quantify the patients’ evolution. Such a questionnaire can be easily used by doctors and surgeons in their daily practice. Indeed, giving a patient permission to return to sport is a difficult decision to make, and there is no consensus on this subject [1]. The purpose of the score is to enable physicians to recognize patients who have psychological factors that prevent them from resuming their activities. Therefore, the practitioners will be able to offer them specific advice to overcome their apprehension.

In this study, the ceiling effect was 19.1%, it has been varied between 6.7 and 33.8%. By recalculating it on the retest values, the value of the ceiling effect is 16%, ranging from 5.7 to 25%; which corresponds to the value found in other studies [5, 13, 17, 28]. These values of the ceiling effect can be explained by the fact that the patients have a median duration of 3 years postoperative, and by the good results of ankle ligament reconstruction [18, 22, 25].

All the patients were operated using the anatomic arthroscopic reconstruction technique. As mentioned previously many different chronic lateral instability surgical techniques exist, and their outcome seems to appear to be similar. This score should be able to be applied to all CLAI surgeries regardless of the method used.

A limitation present in this study was that the model for the ALR-RSI score was not originally developed for ankle instability. Indeed, the score was based on an adaptation of the ACL-RSI score, a validated score to quantify the psychological readiness of athletes to return to sport following surgical ACL reconstruction. Nevertheless, the questions are not specific to a certain articulation of the body, and can easily be transposed to other articulations implicated in sport injuries.

## Conclusion

The ALR-RSI is a valid, reproducible scale to evaluate the relevant psychological factors in the return to the same sport after ankle ligament reconstruction.
